# Master-Leader-Slave Cuckoo Search with Parameter Control for ANN Optimization and Its Real-World Application to Water Quality Prediction

**DOI:** 10.1371/journal.pone.0170372

**Published:** 2017-01-26

**Authors:** Najmeh Sadat Jaddi, Salwani Abdullah, Marlinda Abdul Malek

**Affiliations:** 1 Data Mining and Optimization Research Group (DMO), Centre for Artificial Intelligence Technology, Faculty of Information Science and Technology, Universiti Kebangsaan, Malaysia, Bangi, Selangor, Malaysia; 2 Civil Engineering Department, College of Engineering, Universiti Tenaga Nasional, Jalan IKRAM-UNITEN, Kajang, Selangor, Malaysia; Beihang University, CHINA

## Abstract

Artificial neural networks (ANNs) have been employed to solve a broad variety of tasks. The selection of an ANN model with appropriate weights is important in achieving accurate results. This paper presents an optimization strategy for ANN model selection based on the cuckoo search (CS) algorithm, which is rooted in the obligate brood parasitic actions of some cuckoo species. In order to enhance the convergence ability of basic CS, some modifications are proposed. The fraction *P*_*a*_ of the *n* nests replaced by new nests is a fixed parameter in basic CS. As the selection of *P*_*a*_ is a challenging issue and has a direct effect on exploration and therefore on convergence ability, in this work the *P*_*a*_ is set to a maximum value at initialization to achieve more exploration in early iterations and it is decreased during the search to achieve more exploitation in later iterations until it reaches the minimum value in the final iteration. In addition, a novel master-leader-slave multi-population strategy is used where the slaves employ the best fitness function among all slaves, which is selected by the leader under a certain condition. This fitness function is used for subsequent Lévy flights. In each iteration a copy of the best solution of each slave is migrated to the master and then the best solution is found by the master. The method is tested on benchmark classification and time series prediction problems and the statistical analysis proves the ability of the method. This method is also applied to a real-world water quality prediction problem with promising results.

## Introduction

Computational intelligence is defined as a set of nature-inspired computational approaches to deal with complex real-world problems. This intelligence is directly linked to computing concepts such as fuzzy logic, decision making, artificial neural networks (ANNs), and metaheuristic algorithms as optimization techniques. Artificial neural networks are a family of learning models that are inspired by biological neural networks and are employed to estimate functions that are generally unknown. A number of researchers have used optimization algorithms to train neural network models [1–8]. The multi-layer self-organizing ANN has been studied in the literature and metaheuristic algorithms have been used to optimize the structure of the ANN [9–12]. A few methods have been used to attempt to optimize both the weights and the structure of ANNs [8, 13–15]. Other proposals include the use of an adaptive merging and growing algorithm in the design of ANNs [16] and the adoption of a Taguchi-based parameter for the genetic algorithm used in ANN training [17]. [4]used a pruned probabilistic neural network with a genetic algorithm to optimize the structure of an ANN, while [6] applied particle swarm optimization (PSO) to optimize an ANN. Other PSO based approaches can be found in [18, 19]. In another research study [20], the authors used a combination of self-organizing networks and an artificial immune system to minimize the neurons in an ANN.

Finding the global optimum solution is the major and common aim of optimization algorithms. Good communication between diverse exploration and intensive exploitation results in good convergence in any algorithm. There is no set method for achieving a balance between exploration and exploitation. Different methods have been proposed for different algorithms to achieve this trade-off. Based on a review of the relevant literature, the obvious solution is that more efforts should focus on improving the diverse exploration phase in early iterations and on enhancing intensive exploitation in later iterations so that discovery of the global optimum will most likely be achieved.

Many research studies have found that the use of a multi-population in the algorithm results in more exploration and enables the algorithm to move toward the global optimum. For instance, the authors in [21] proposed a multi-population cooperative method for PSO, called CPSO-S, in which the solution vector is split into smaller sub-vectors. Each of these sub-vectors is optimized using a separate swarm. A complete solution vector is built by using the best solution found by each swarm. Another study in [22] proposed a master-slave multi-population for PSO. Other works based on PSO include [23–26]. Other works based on PSO include Yen and Daneshyari [23], Zhan and Zhang [24], Zhang, Cui and Zou [25], and El-Abd and Kamel [26]. A multi-population cooperative method for bee swarm optimization has also been studied [27]. [28] proposeda multi-population cultural algorithm, in which the competitive multi-population genetic algorithm is embedded into the population of the cultural algorithm. Recently, a multi-population cooperative bat-inspired algorithm for optimization of the ANN model has also been proposed [29].

In this paper, we propose an optimization methodology based on the cuckoo search (CS) algorithm. Cuckoo search is rooted in the obligate brood parasitic actions of some cuckoo species. The key advantage of the CS algorithm is its simplicity. Unlike other population-based algorithms there is only one parameter, *P*_*a*_, in CS, which makes it easy to implement. A further advantage of CS is that its search uses Lévy flights instead of standard random walks; Lévy flights include infinite mean and variance so can explore the search space more efficiently compared to random walks. The CS algorithm was first proposed by [30] and its superior ability was quickly established in many areas of optimization [31–37].

To enhance the performance of the CS algorithm and its convergence ability, we perform the following modifications. First, the fraction *P*_*a*_ of the *n* nests replaced by new nests is a parameter that is fixed in basic CS. As the selection of *P*_*a*_ has a direct effect on exploration in the search space, in the proposed method *P*_*a*_ is set to a maximum value at initialization to achieve more exploration or diversification in early iterations and it is decreased during the search to achieve more exploitation or intensification in later iterations until it reaches the minimum value in the final iteration. This first modification gives the algorithm fewer parameters to work with and also makes it a self-adaptive algorithm. Furthermore, the modification enhances the algorithm’s convergence ability by improving the balance between exploration and exploitation. The second modification involves the application of a novel master-leader-slave multi-population strategy, where the slaves employ the best fitness function among all slaves which is selected by the leader unit for the next Lévy flight if, after a certain number of iterations, there is no improvement in the quality of the best solution. In each iteration a copy of the best solution of each slave is migrated to the master and then the best solution among these is found by applying CS to the master sub-population. In this strategy, exploration is provided by the slaves and exploitation is achieved by the master.

The rest of this paper is organized as follows: this paper firstly provides a brief description of cuckoo behavior and then introduces the mechanism of the CS algorithm and Lévy flights, followed by the explanation on the proposed modifications of the basic CS algorithm. After that, the experimental results of applying the proposed approach on benchmark datasets and on real-world water quality data are given. The conclusion of this work is provided in last section.

## Reproductive Behaviour of Cuckoos

Some cuckoo species lay their eggs in the nest of another bird species alongside the host bird’s eggs, while others remove the host eggs and then lay their own in the nest to increase the hatching probability of their own eggs [30]. Female parasitic cuckoos are specialized in mimicking the pattern and color of the eggs of a few selected host species. This decreases the likelihood of the eggs being abandoned and so improves the reproductive outcome for the cuckoo. However, if a host bird discovers that the eggs are not its own, then it either throws them away or just abandons its nest and makes a new nest elsewhere. The parasitic cuckoo often selects a nest anywhere the host bird has just laid its eggs. Generally, the cuckoo eggs hatch a little before the host bird’s eggs. When the first cuckoo chick is hatched, its first natural action is to throw out the host eggs by blindly pushing the eggs out of the nest. This behavior has the effect of increasing the cuckoo chicks’ share of the food provided by the host bird. A cuckoo chick can also mimic the call of the host chicks to increase its chances of being fed.

## Cuckoo Search Algorithm

The first version of the CS algorithm was introduced by [30]. The authors made three idealized assumptions to ensure the simplicity of the CS algorithm. In this simplified form:

- Each cuckoo lays one egg at a time in a random nest;
- The best nests containing high-quality eggs are kept for the next iteration;
- The number of on-hand host nests is fixed and the probability of the egg laid by the cuckoo being discovered by the host bird is considered as *P*_*a*_ ϵ [0, 1]. If the cuckoo egg is found, the host bird can either discard the egg or leave the nest and build a new nest in a new place. This assumption is simulated by a fraction of *P*_*a*_ of *n* nests being replaced by new nests (having new random solutions).

In this simple form of CS each egg in a nest stands for one solution, and a cuckoo egg corresponds to a new solution. In this algorithm the aim is to replace a poor-quality solution in the nest with new and possibly better solutions.

In the CS algorithm a new solution is generated by using Lévy flights. ALévy flight is a kind of random walk in which the random step lengths have a probability distribution. The reason for using Lévy flights in basic CS is that it is more efficient for exploration of the search space compared to random walk. This efficiency is due to the longer step length in Lévy flights. In the CS algorithm the Lévy flights are performed as shown by Eq 1:

$$x_{i}^{\left(t+1\right)}=x_{i}^{\left(t\right)}+\beta ⊕\text{Lévy}(\lambda )$$ (1)

where *β* > 0 is denoted as the step size. The step size depends on the scale of the problem. For most problems the value of *β* can be set to 1 [30]. The symbol ⊕ denotes entrywise multiplication. The Lévy flights provide a random walk with a random step length, which is derived from a Lévy distribution that contains infinite variance with infinite mean, as expressed by Eq 2:

$$\text{Lévy}\approx \text{u}={\text{t}}^{-\lambda },1<\lambda \leq 3$$ (2)

The idea of using Lévy flights originated in the cuckoo’s reproductive behavior; if a cuckoo’s egg appears similar to a host’s eggs, the probability of the cuckoo’s egg being discovered by the host bird is less. Therefore, in the simulation of the CS algorithm, the differentiation of the solutions using Lévy flights is used in generating new solutions for the CS algorithm.

**Fig 1:**
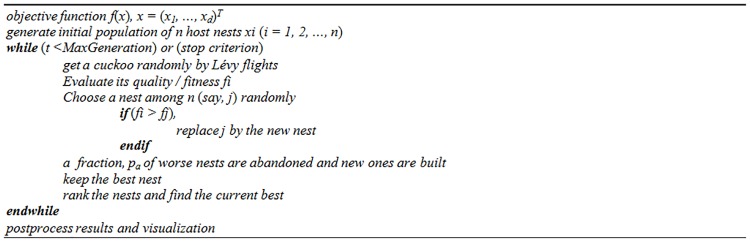
Pseudocode of basic CS algorithm.

Similar to other population-based algorithms, the CS starts with an initial random population. In the main loop of this algorithm, when the stopping criterion is not met the algorithm will get a cuckoo (new solution) by Lévy flights, and if its fitness is better than a random solution in the population then the random solution is replaced by the new solution. The worst solutions are abandoned and new random solutions are replaced under the condition of a fraction of *P*_*a*_. In the final step of each iteration the solutions are ranked and the best one is updated. The pseudocode to maximize the result of the basic CS taken from [30] is shown in Fig 1.

## Modified Cuckoo Search Algorithm

We propose two modifications of CS to improve the performance of the basic CS. The first modification aims to enhance the overall performance of CS by controlling the *P*_*a*_ parameter. Controlling this parameter improves the balance between exploration and exploitation of the search space and can therefore increase the likelihood of fast convergence to the global optimum. The second modification involves maintaining the diversity of the solutions and directing the search process toward the best solution by using a novel master-leader-slave multi-population strategy. The details of these modifications are discussed in the following subsections.

### *P*_*a*_ parameter control

The main idea of the CS is based on decreasing the probability of the cuckoo’s eggs being detected by the host bird so the cuckoo’s eggs have more opportunity to survive and become mature cuckoos. Therefore, in this algorithm if more eggs survive it means that the algorithm is going to optimize and converge toward the best solution. The *P*_*a*_ parameter is a very important parameter in adjusting the convergence rate of the algorithm. In the basic CS algorithm, the fixed value of the *P*_*a*_ parameter is set in the initialization step and cannot be altered during the search process. The main challenge encountered in the basic CS algorithm is how to tune this parameter to find the global optimum solution. If the value of *P*_*a*_ is small the probability of the host bird detecting the cuckoo’s egg is low. In this case there is an insufficient diversity of solutions and therefore there is not enough exploration of the search space. Inadequate exploration might decrease the performance and result in finding a poor solution. If the value of *P*_*a*_ is large the probability of the host bird detecting the cuckoo’s egg is high. So there is high exploration but there is not enough exploitation to converge the algorithm toward the optimum solution.

We propose a simple yet effective way to fine-tune the *P*_*a*_ parameter to overcome this challenging issue. In order to improve the performance of the algorithm, the value of *P*_*a*_ must be big enough in the early iterations to force the algorithm to maintain the diversity of the solutions and increase exploration of the search space. However, the value of *P*_*a*_ should be decreased in later iterations to speed up the convergence of the algorithm. In the proposed modified CS algorithm, the value of the *P*_*a*_ parameter is changed dynamically with the number of iterations. Since *P*_*a*_ ϵ [0, 1] in basic CS, the maximum value in this range, which is equal to 1, is considered for *P*_*a*_ in the initial setting and then it is decreased in each iteration at a rate that is calculated by Eq 3 until it reaches the minimum value of 0 in the range of [0, 1]:

$$DR_{i}^{P_{a}}=1-\frac{iteration_{i}}{numOfIte}$$ (3)

where the decreasing rate of *P*_*a*_ in the *i*^*th*^ iteration is denoted as $DR_{i}^{P_{a}}$, the number of the current iteration is indicated as *iteration*_*i*_, and *numOfIte* stands for the total number of iterations.

**Fig 2:**
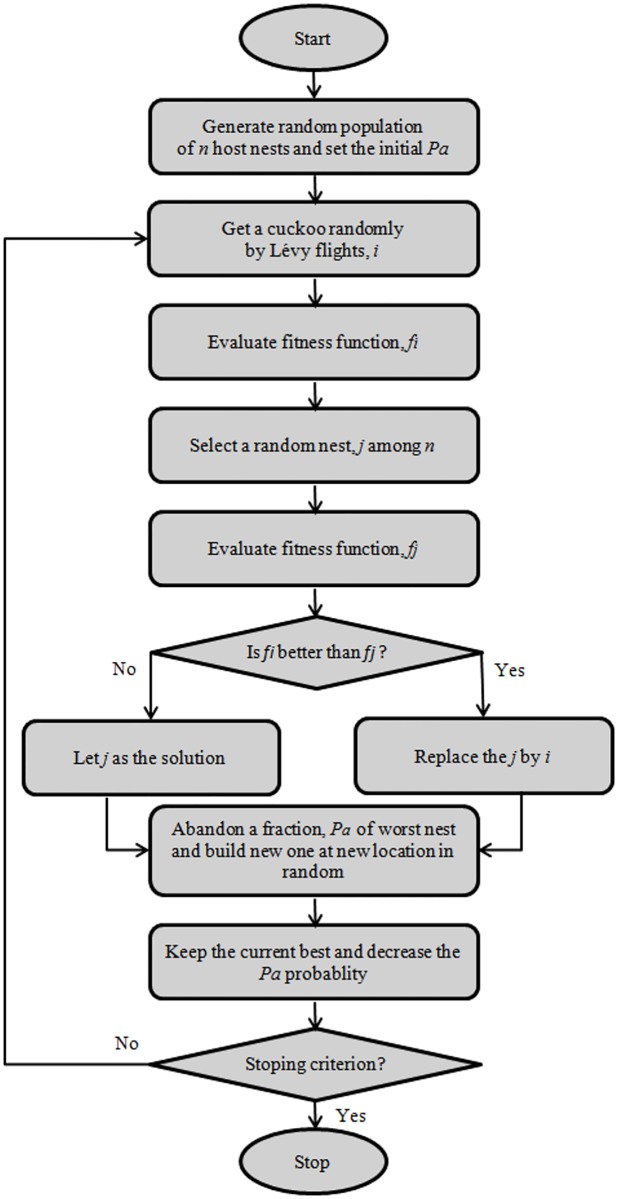
Flowchart of CS algorithm showing *P*_*a*_ parameter control.

This parameter control strategy reduces the parameters as the *P*_*a*_ parameter is automatically changed during the search process based on the number of the current iteration and the total number of iterations. The schematic flowchart of the modified CS algorithm with *P*_*a*_ control is illustrated in Fig 2.

### Master-leader-slave multi-population

In multi-population cooperative PSO [38], a master-slave approach is used that employs one master swarm and a number of slave swarms. The slave swarms apply the PSO algorithm in parallel and then each transfers its best solution to the master. Then the master updates the best solution from among all the slaves’ best solutions. A modified master-slave multi-population, which is a combination of the ring and master-slave for the bat algorithm was proposed in our earlier work [29]. In that work the slaves exchange information on their best solutions in a ring-like manner if, after a certain number of iterations, there is no improvement on the best solution. Further cooperation is achieved by sending the best solution of each slave to the master in each iteration. The master involves the application of an optimization algorithm on the population of best solutions collected from all the slaves. The advantage of this work lies in its ability to maintain the diversity of the solutions, where exploration of the population is conducted by the slaves and exploitation is performed by the master.

**Fig 3:**
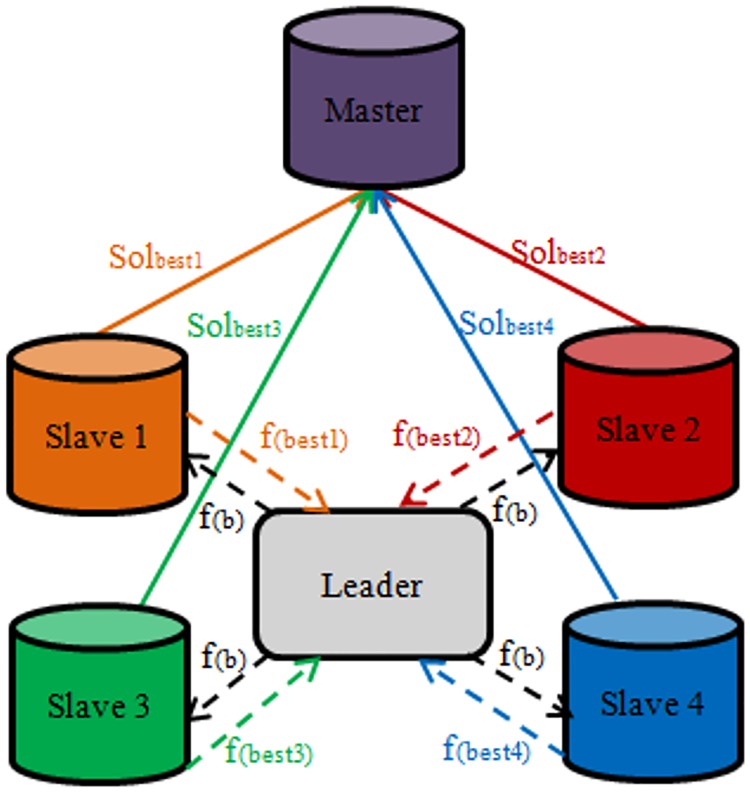
Schematic of master-leader-slave strategy.

We take this approach one step further to propose a novel multi-population cooperative strategy called master-leader-slave for CS, which is based on the master-slave strategy with the addition of another unit called the leader. The leader unit does not involve the use of any optimization procedure but it receives the best solutions found by the slaves if, after a certain number of iterations, there is no improvement in the quality of the best solution. After receiving the best solutions from the slaves the leader selects the best solution from among all the best solutions from the slaves. Then, the leader sends the information on the selected solution to all the slaves to guide them to follow this selected solution in the next Lévy flight. The master receives a copy of the best solutions found by the slaves in each iteration and applies the CS optimization algorithm to the population of best solutions from the slaves. Then the overall best solution is updated and the master is zeroized. The cooperation between slaves, leader and master is shown schematically in Fig 3.

**Fig 4:**
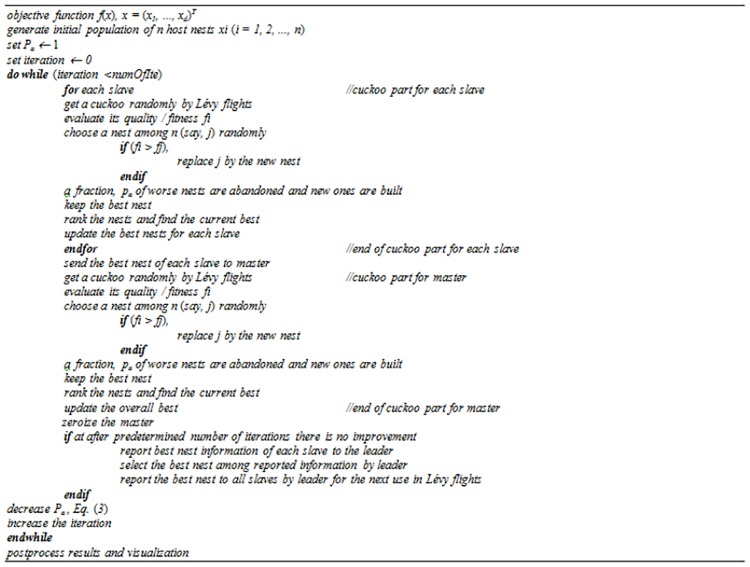
Pseudocode of CS algorithm with *P*_*a*_ parameter control and multi-population.

In Fig 3, the arrows labeled Sol_best_ show that a copy of the best solution is transferred to the master. While the arrows labeled f_(best)_ denote that the information (quality) about the best solution is sent to the leader. The arrows labeled f_(b)_ illustrate that the information on the best solution among all the best solutions from the slaves is sent to the slaves. The cooperation between slaves and leader gives the algorithm a powerful exploration capability and provides a high diversity of solutions in the population, while the support given by the master to the slaves improves the ability of algorithm to achieve fast convergence. This proposed multi-population cooperative strategy is applied to improve the performance of the basic CS algorithm. The pseudocode of the proposed algorithm with modifications is shown in Fig 4.

## Experimental Results

For the ANN used in this present study, two hidden layers with two nodes for each hidden layer were selected as this format is commonly used and is the most accurate [8, 29]. The activation function used in this experiment was the hyperbolic tangent as it has better presentation [39] than other activation functions. A one-dimensional vector was used for the solution representation, where the weights and biases of the ANN are located in each cell of this vector. The length of the vector is equivalent to the number of weights plus the number of biases of the ANN.

### Benchmark classification and time series prediction problems

In this section, we examine the performance of the basic CS algorithm (CS), the proposed modified CS algorithm with *P*_*a*_ control (*P*_*a*_Ctrl-CS) and the proposed modified CS algorithm with parameter control and multi-population (Multipop-*P*_*a*_Ctrl-CS) by applying them to six classification and two standard time series prediction problems. The classification problems are Iris, Diabetes diagnoses, Thyroid dysfunction, Breast cancer, Credit card, and Glass identification. The time series prediction problems are Mackey-Glass and Gas Furnace; the former is a univariate dataset, whereas the latter is a multivariate dataset. The classification problems are taken from the UCI machine learning repository [40]. The Gas Furnace dataset is available from http://datasets.connectmv.com/datasets, while Mackey-Glass was produced from equation in the literature [29]. The characteristics of the datasets are: Iris dataset with 150 examples, four features and three classes; Diabetes dataset with 768 examples, eight features and two classes; Thyroid dataset with 7200 examples, 21 features and three classes; Cancer dataset with 699 examples, 10 features and two classes; Card dataset with 690 examples, 15 features and two classes; Glass dataset with 214 examples, 10 features and six classes as classification datasets and Mackey-Glass dataset with 1000 examples and one feature along with Gas Furnace dataset with 296 examples and two features as time series prediction datasets.

The initial parameters are shown in Table 1. The values of *α* and *P*_*a*_ in the basic CS algorithm are adopted from [30], while the value of *P*_*a*_ in the modified CS algorithms is changed within the range of [0, 1]. This range is based on the suggestion in [30]. The ANN consists of two hidden layers with two nodes for each hidden layer as this structure has been used in previous related research [8, 29]. The activation function for this experiment is the hyperbolic tangent as it has superior performance compared to other activation functions [39]. The solutions are represented as a one-dimensional vector, where the weights and biases of the ANN are placed in each cell of this vector. The length of the vector is equal to the number of weights plus the number of biases of the ANN.

**Table 1:** Configuration of parameters.

Parameter	Definition	Value
*popSize*	Size of population	100
*numOfIte*	Maximum number of iterations	100
*Α*	Step size	1
*P*_*a*_ (in basic CS)	The fraction of the nests replaced by new nests	0.25
*P*_*a*_ (in modified CSs)	The fraction of the nests replaced by new nests	[0, 1]

We considered the output of *x*(*t*+6) with the input variables of *x*(*t*), *x*(*t*-6), *x*(*t*-12) and *x*(*t*-18) for the Mackey-Glass dataset. For the Gas Furnace problem, the input variables were *u*(*t*-3), *u*(*t*-2), *u*(*t*-1), *y*(*t*-3), *y*(*t*-2), *y*(*t*-1) and the output variable was *y*(*t*), as used in earlier works. We used 30 twofold iterations [8] to evaluate the performance of the model. The data were randomly separated into two parts for each run. One half was used for the training set and the other half was employed as the testing set to test the model. The examples in the datasets were normalized into the range of [–1, 1]. We compare the results in the following two subsections. The first investigates the performance of the proposed algorithms in comparison with each other and the second presents a comparison of the best proposed method with the approaches in the literature.

#### Results of comparison of proposed methods

In this section, we evaluate the performance of the basic CS and the two proposed methods based on the percentage of the error. A summary of the results obtained by the three versions of the algorithm is shown in Table 2. In the case of the classification datasets (the first six datasets in the table) the training and testing errors are represented by the classification error. In the case of the Mackey-Glass time series dataset the training and testing error is represented by the root mean squared error (RMSE), whereas for the Gas Furnace time series dataset it is denoted by the mean square error (MSE). From Table 2 it can be seen that Multipop-*P*_*a*_Ctrl-CS has fairly superior performance compared to the other methods. To confirm the above finding we carried out an average ranking test to discover the first-ranked algorithm. The results are shown in Table 3, from which it can be seen that Multipop-*P*_*a*_Ctrl-CS is ranked first in two cases for training error and testing error and *P*_*a*_Ctrl-CS and CS are ranked second and third, respectively.

**Table 2:** Results for the three versions of the CS algorithm.

Dataset	Criteria	CS	*P*_*a*_Ctrl-CS	Multipop-*P*_*a*_Ctrl-CS
Iris	Training error %	2.4213	1.8522	1.5311
	Std. Dev.	0.0645	0.0789	0.0213
	Testing error%	2.6072	2.0756	1.6906
	Std. Dev.	0.0456	0.0460	0.0122
Diabetes	Training error %	16.8243	16.5118	13.9689
	Std. Dev.	0.0435	0.0585	0.0345
	Testing error%	21.8243	20.1785	15.6356
	Std. Dev.	0.0579	0.0042	0.0294
Thyroid	Training error %	6.5545	4.2117	4.477
	Std. Dev.	0.0041	0.0041	0.0041
	Testing error%	7.2545	6.7117	5.1104
	Std. Dev.	0.0456	0.0786	0.0159
Cancer	Training error %	2.7280	2.5682	2.3596
	Std. Dev.	0.0045	0.0022	0.0056
	Testing error%	3.1614	2.8349	2.4596
	Std. Dev.	0.0517	0.0325	0.0480
Card	Training error %	13.5381	12.9943	12.5747
	Std. Dev.	0.0459	0.0284	0.0739
	Testing error%	14.4715	13.7343	12.5831
	Std. Dev.	0.0027	0.0083	0.0064
Glass	Training error %	34.7632	25.7255	26.0586
	Std. Dev.	0.0043	0.0065	0.0328
	Testing error%	41.4299	38.7255	28.0919
	Std. Dev.	0.0836	0.0111	0.0566
Mackey-Glass	Training error %	2.6E-03	2.5E-03	1.4E-03
	Std. Dev.	0.0001	0.0001	0.0001
	Testing error%	2.9E-03	2.9E-03	1.4E-03
	Std. Dev.	4.6E-06	0.0001	0.0001
Gas Furnace	Training error %	0.3909	0.4085	0.1837
	Std. Dev.	5.0E-06	3.0E-06	4.0E-06
	Testing error%	0.4343	0.4618	0.1903
	Std. Dev.	0.0072	0.0067	0.0023

**Table 3:** Average rankings for the three versions of the CS algorithm.

Training error		Testing error	
Algorithm	Rank	Algorithm	Rank
Multipop-*P*_*a*_Ctrl-CS	1.1111	Multipop-*P*_*a*_Ctrl-CS	1. 25
*P*_*a*_Ctrl-CS	2	*P*_*a*_Ctrl-CS	2
CS	2.7778	CS	2.75

**Fig 5:**
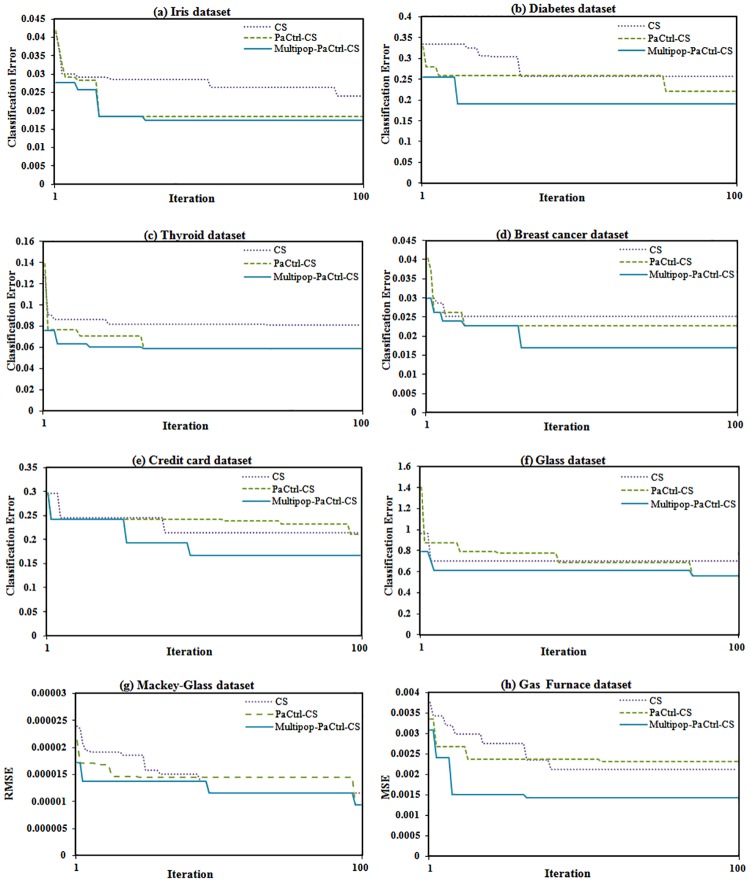
Comparison of optimization progress of CS, *P*_*a*_Ctrl-CS and Multipop-*P*_*a*_Ctrl-CS: (a) Iris dataset, (b) Diabetes dataset, (c) Thyroid dataset, (d) Breast cancer dataset, (e) Credit card dataset, (f) Glass dataset, (g) Mackey- Glass dataset, and (h) Gas Furnace dataset.

In order to further investigate the performance of proposed methods, a comparison of the optimization progress of CS, *P*_*a*_Ctrl-CS and Multipop-*P*_*a*_Ctrl-CS was conducted, the results of which are provided in Fig 5. This figure shows the optimization progress of the proposed methods in 100 iterations for all tested datasets. In all cases Multipop-*P*_*a*_Ctrl-CS starts with a better solution and also converges to a better solution compared to CS and *P*_*a*_Ctrl-CS. This is because the multi-population has superior exploration and guides the search toward the global optimum by using the leader and master actions of the master-leader-slave strategy. From Fig 5 it is also evident that *P*_*a*_Ctrl-CS performs better than CS in most cases. This is because the method is designed to control the *P*_*a*_ parameter during the search process to improve exploration in early iterations and to achieve more exploitation in later iterations until final convergence is reached.

To measure whether Multipop-*P*_*a*_Ctrl-CS is statistically different from the other proposed methods, we computed the *p*-values of the three algorithms for all datasets, where the critical value *α* is equal to 0.05. Critical values for a test of hypothesis depend upon a test statistic, which is specific to the type of test, and the significance level, *α*, which defines the sensitivity of the test. A value of *α* = 0.05 implies that the null hypothesis is rejected 5% of the time when it is in fact true. The most commonly used significance level is *α* = 0.05.

This evaluation was carried out for the training error and testing error. The results are shown in Table 4. Values lower than the critical level (highlighted in bold) confirm the superior ability of Multipop-*P*_*a*_Ctrl-CS. Only in the case of four training errors where we compare *P*_*a*_Ctrl-CS andMultipop-*P*_*a*_Ctrl-CS is the *p*-value higher than the critical value. This proves the superior performance of Multipop-*P*_*a*_Ctrl-CS compared to *P*_*a*_Ctrl-CS and CS.

**Table 4:** Pairwise comparison of *p*-values of Multipop-*P*_*a*_Ctrl-CSwith those of CS and *P*_*a*_Ctrl-CS.

Dataset	CS		*P*_*a*_Ctrl-CS	
	Training error	Testing error	Training error	Testing error
Iris	**6.2E-06**	**0.0005**	**0.0003**	**0.0017**
Diabetes	**0.0258**	**6.9E-08**	**0.0368**	**5.4E-05**
Thyroid	**0.0015**	**8.1E-06**	0.3879	**0.0002**
Cancer	**0.0360**	**0.0002**	0.1381	**0.0070**
Card	**0.0026**	**2.7E-10**	0.0541	**6.1E-05**
Glass	**0.0026**	**7.5E-07**	0.4500	**1.2E-05**
Mackey-Glass	**1.7E-05**	**5.2E-06**	**1.4E-05**	**3.0E-06**
Gas Furnace	**4.1E-07**	**1.7–09**	**9.5E-08**	**1.2E-10**

#### Results of comparison of the best proposed method with other methods in the literature

There are plentiful studies on the ANN, particularly in relation to its application to classification problems. Therefore, we compared Multipop-*P*_*a*_Ctrl-CS as the best method among those examined in the previous section with the most recent methods in the literature that are mainly related to our proposed method and that employed the same datasets. Comparisons of the testing errors for classification and time series prediction are reported in Tables 5 and 6, respectively. The best results in these tables are shown in bold.

**Table 5:** Comparison of Multipop-*P*_*a*_Ctrl-CS and other methods for the classification problem.

Dataset	Multipop-*P*_*a*_Ctrl-CS	BatRM-S	SA	TS	GA	TSa	GaTSa+BP	PSO
Iris	1.6906	**1.5309**	12.649	12.478	2.5641	4.6154	5.2564	3.8341
Diabetes	**15.6356**	19.3021	27.156	27.404	25.994	25.876	27.061	22.742
Thyroid	**5.1104**	6.2435	7.3813	7.3406	7.2850	7.3322	7.1509	5.4328
Cancer	2.4596	2.9928	7.1729	7.2779	7.4220	6.2846	7.1920	1.9582
Card	**12.5831**	13.4163	23.469	18.042	31.724	21.269	15.242	13.239
Glass	**28.0919**	36.4251	58.381	56.412	58.031	57.777	55.142	27.823

**Table 6:** Comparison of Multipop-*P*_*a*_Ctrl-CS and other methods for the time series prediction problem.

Dataset	Multipop-*P*_*a*_Ctrl-CS	BatRM-S	gHFSNN (triangular)	gHFSPNN (Gaussian)	gFSPNNT*(triangular)	gFSPNNT*(Gaussian)	gFSPNNT(triangular)	gFSPNNT(Gaussian)	gFPNN(Triangular)	gFPNN(Gaussian)	PSO
Mackey-Glass	**0.0014**	0.0017	0.0315	0.0262	0.0441	0.0289	0.1180	0.0441	-	-	0.0016
Gas Furnace	**0.1903**	0.3408	11.520	10.250	-	-	11.200	10.300	10.300	10.000	0.2739

Note: The percentage of error has been calculated for the results in the literature.

“*” is a part of method’s name as presented in the original paper.

Table 5 illustrates the results of a comparison of Multipop-*P*_*a*_Ctrl-CSwithseven methods, namely, the multi-population of the bat algorithm (BatRM-S) [29], simulated annealing (SA), tabu search (TS), genetic algorithm (GA), combination of TS and SA (TSa), and integration of TS, SA and GA and backpropagation (GaTSa+BP) [8] and standard particle swarm optimization (PSO) which was re-implemented to be compared with proposed method for the classification problem. Table 5 shows that Multipop-*P*_*a*_Ctrl-CS exhibits higher performance than the other methods in the literature. The Multipop-*P*_*a*_Ctrl-CS algorithm outperforms these methods in four out of the six tested methods.

Table 6 provides the results of a comparison of Multipop-*P*_*a*_Ctrl-CSwith several algorithms investigated in [10, 11, 29]and re-implemented PSOfor the time series prediction problem. For both the Mackay-Glass and Gas Furnace time series datasets Multipop-*P*_*a*_Ctrl-CS achieved the best results compared to the other approaches in the literature. The superiority of Multipop-*P*_*a*_Ctrl-CS is due to its ability to control the *P*_*a*_ parameter to provide better exploration and enhanced exploitation during the search process as well as to its use of master-leader-slave multi-population strategy.

In order to further validate the results, we carried out a Friedman test and a Nemenyi test on Multipop-*P*_*a*_Ctrl-CS. These tests were used to determine whether there are significant differences between the achievement of the method and the other methods in the literature in terms of classification error and prediction error.

In the case of classification problems, the Friedman test result was 28.1625, which is greater than 13.45 (critical value) for the testing error of classification problems. The critical value 13.45 was found in table of critical values for the Chi-Square test where the degree of freedom is equal to *K*-1. *K* is the number of methods, which in our experiment is equal to eight. Since the value of the Friedman test was greater than the critical value, the null hypothesis was rejected. This evaluation showed that there is a significant difference in performance between the algorithms in terms of classification error.

The Nemenyi test was also carried out as a post-hoc test to discover the group of methods that are differ from the other methods. The standard error (SE) was calculated and its posterior computing the minimum significant difference (MSD) was computed. The value of the MSD is calculated to see where any differences in averages were higher than the MSD. The MSD in our experiment was equal to 7.579328. The result of the Nemenyi test is highlighted in bold in Table 7. This table shows that Multipop-*P*_*a*_Ctrl-CS has a statistically significant difference in six cases.

**Table 7:** Nemenyi test results for classification error.

		Multipop-*P*_*a*_Ctrl-CS	BatRM-S	SA	TS	GA	TSa	GaTSa+BP	PSO
	mean	10.92853	13.31845	22.70153	21.49242	22.17002	20.5257	19.50738	**12.50485**
Multipop-*P*_*a*_Ctrl-CS	10.92853	-	2.389917	**11.77300**	**10.56388**	**11.24148**	**9.597167**	**8.578850**	**9.232193**
BatRM-S	13.31845	-	-	**9.383083**	**8.173967**	**8.851567**	7.20725	4.739600	**8.827967**
SA	22.70153	-	-	-	1.209117	0.531517	2.175833	**17.96193**	7.483735
TS	21.49242	-	-	-	-	0.677600	0.966717	3.530483	6.368425
GA	22.17002	-	-	-	-	-	1.644317	**18.63953**	**11.72492**
TSa	20.5257	-	-	-	-	-	-	1.886167	3.349553
GaTSa+BP	19.50738	-	-	-	-	-	-	-	7.137142
PSO	12.50485	-	-	-	-	-	-	-	-

The Friedman test result for the time series prediction was equal to 9.3374. This value is larger than the critical level (9.143), so we rejected the null hypothesis. We also performed a Nemenyi post-hoc test for time series prediction and the results are shown in Table 8. The MSD is equal to 4.209448 and from the highlighted values in Table 8 it can be seen that Multipop-*P*_*a*_Ctrl-CS performed better in four cases.

**Table 8:** Nemenyi test results for prediction error of time series prediction.

Algorithm		Multipop-*P*_*a*_Ctrl-CS	BatRM-S	gHFSPNN(triangular)	gHFSPNN(Gaussian)	gFSPNN T(triangular)	gFSPNN T(Gaussian)	PSO
	Mean	0.09585	0.17125	5.77575	5.1381	5.659	5.17205	0.2755
Multipop-*P*_*a*_Ctrl-CS	0.09585	-	0.07540	**5.67990**	**5.04225**	**5.56315**	**5.07620**	**4.24335**
BatRM-S	0.17125	-	-	**5.60450**	**4.96685**	**5.48775**	**5.00080**	**5.27349**
gHFSPNN(triangular)	5.77575	-	-	-	0.63765	0.11675	0.60370	0.34834
gHFSPNN(Gaussian)	5.1381	-	-	-	-	0.52090	0.03395	0.23846
gFSPNNT(triangular)	5.659	-	-	-	-	-	0.48695	0.83932
gFSPNN T(Gaussian)	5.17205	-	-	-	-	-	-	0.98478
PSO	0.2755	-	-	-	-	-	-	-

### Real-world application of proposed methods to water quality prediction

In the last part of our study, the proposed methods were applied to real-world water quality data. The data were collected from a weather station near Kajang in the Selangor area of Malaysia. The data comprises monthly water quality data records from the years 2004 through 2013. They are multivariate time series and have been used as a prediction problem. The data contain 13 features: SFLOW, TEMP (Degrees C), TUR (NTU), DS (mg/l), TS (mg/l), NO_3_ (mg/l), PO_4_ (mg/l), DO (mg/l), BOD (mg/l), COD (mg/l), SS (mg/l), pH (unit), NH_3_-NL (mg/l).

All the features were employed as input for the ANN and the last six features were considered as output of the ANN as they are the most critical features for water quality prediction. The data were divided into two parts; 70% of the data was used as a training set and 30% was used as a testing set. The data were normalized into the range of (0, 1) using the Min-Max normalization technique. A 10-fold cross-validation was used to validate the results. One step ahead prediction was performed. The averages of 30 runs for the prediction are shown in Table 9. For ease of reference and assessment, the average ranking of the training and testing errors of the proposed algorithm are provided in Table 10. As shown in this table, Multipop-*P*_*a*_Ctrl-CS is ranked first for both training and testing errors.

**Table 9:** Prediction results of Multipop-*P*_*a*_Ctrl-CS for real-world water quality data.

Feature	Criteria	CS	*P*_*a*_Ctrl-CS	Multipop-*P*_*a*_Ctrl-CS
NH_3_-NL	Training error	0.5242	0.3185	0.2153
	Testing error	0.5260	0.3207	0.2169
	Predicted value	0.2436	0.3293	0.3823
PH	Training error	0.3242	0.2185	0.1153
	Testing error	0.3260	0.2207	0.1169
	Predicted value	0.5258	0.6483	0.7267
SS	Training error	0.4242	0.2685	0.1653
	Testing error	0.4260	0.2707	0.1669
	Predicted value	0.2457	0.2457	0.1878
COD	Training error	0.4342	0.3385	0.3153
	Testing error	0.4560	0.3407	0.3169
	Predicted value	0.1348	0.2004	0.1772
BOD	Training error	0.3342	0.3485	0.1153
	Testing error	0.3560	0.1407	0.1169
	Predicted value	0.3472	0.3391	0.2436
DO	Training error	0.3842	0.2485	0.1453
	Testing error	0.3860	0.2407	0.1569
	Predicted value	0.2567	0.7618	0.5654

**Table 10:** Average of rankings of the three versions of the CS algorithm for real-world water quality data.

Training error		Testing error	
Algorithm	Rank	Algorithm	Rank
Multipop-*P*_*a*_Ctrl-CS	1	Multipop-*P*_*a*_Ctrl-CS	1
*P*_*a*_Ctrl-CS	2.8333	*P*_*a*_Ctrl-CS	2
CS	2.1666	CS	3

To determine whether there is significant difference between the results of Multipop-*P*_*a*_Ctrl-CS, *P*_*a*_Ctrl-CS and basic CS, the *p*-values were calculated and compared. The results are shown in Table 11. From the table it can be seen that all the *p*-values are much lower than the critical level of 0.05, which proves the higher performance of Multipop-*P*_*a*_Ctrl-CS compared to *P*_*a*_Ctrl-CS and CS.

**Table 11:** Pairwise comparison of *p*-values of Multipop-*P*_*a*_Ctrl-CS with those of CS and *P*_*a*_Ctrl-CSfor real-world water quality data.

Dataset	CS		*P*_*a*_Ctrl-CS	
	Training error	Testing error	Training error	Testing error
NH_3_-NL	**3.19E-46**	**3.49E-41**	**3.67E-41**	**9.21E-37**
PH	**2.66E-41**	**2.82E-36**	**3.67E-41**	**3.67E-41**
SS	**5.32E-44**	**5.76E-39**	**3.67E-41**	**9.21E-37**
COD	**3.15E-34**	**3.55E-31**	**1.38E-22**	**1.20E-18**
BOD	**6.86E-42**	**5.87E-38**	**2.03E-51**	**1.20E-18**
DO	**5.46E-43**	**2.02E-37**	**3.67E-41**	**4.42E-34**

**Fig 6:**
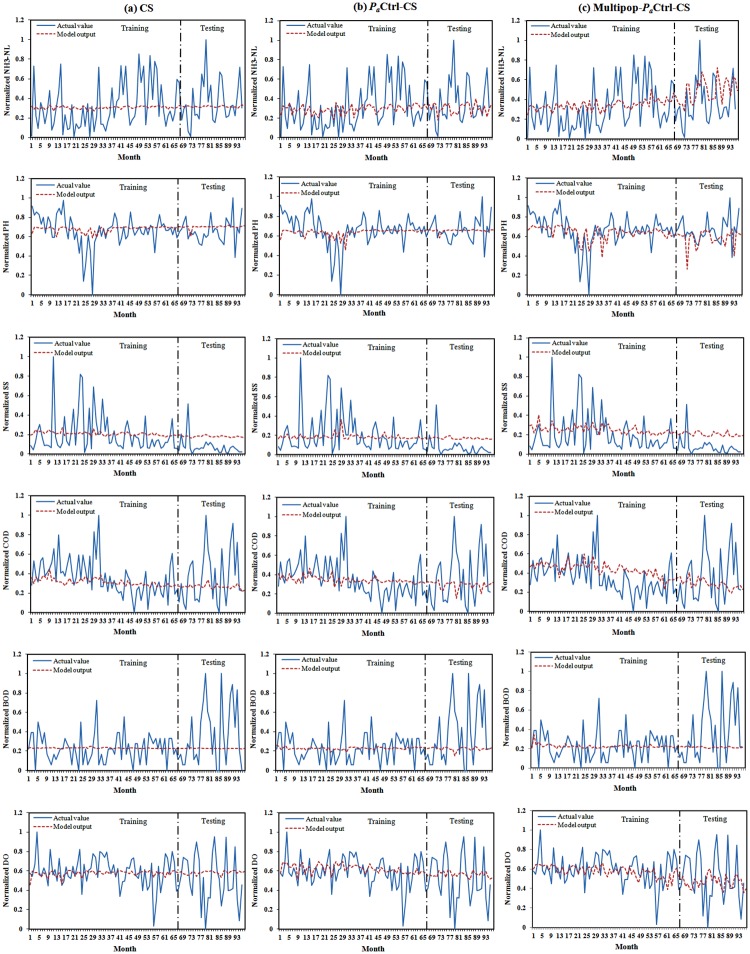
Comparison of actual value and model output for different features in: (a) CS,(b) *P*_*a*_Ctrl-CS and (c) Multipop-*P*_*a*_Ctrl-CS.

A comparison of the actual value and predicted value in both the training and testing parts of for CS, *P*_*a*_Ctrl-CS, and Multipop-*P*_*a*_Ctrl-CS when tested on real-world water quality data are shown in Fig 6. The first column in this figure (Fig 6(a)) provides the results for CS, the second column (Fig 6(b)) shows the results for *P*_*a*_Ctrl-CS, and the last column (Fig 6(c)) illustrates the results for Multipop-*P*_*a*_Ctrl-CS. It is clear that, although the data have an irregular pattern and high fluctuation, the ability of Multipop-*P*_*a*_Ctrl-CS to predict the features is better than the other two methods and all three are acceptable for application to real-world data prediction problem.

The predicted parameters can be used for the Water Quality Index (WQI) calculation and to find the degree of water quality. It means that the WQI can be derived using NH3-NL, PH, SS, COD, BOD, and DO, which are predicted by the proposed method. These parameters represent significant chemical, physical and biological parameters of water quality conditions. Using WQI, numerical face defining certain level of water quality can be presented. Therefore, WQI summarizes water quality data into a simple concept (like a grade) such as excellent in the range of (90, 100), good in (70, 89), medium in (50, 69), bad in (25, 49) and very bad in the range of (0, 24) in a reliable way [41].

## Conclusion

This paper examined the capability of the cuckoo search algorithm and its modifications to contribute to a more accurate ANN model. To attain this important aim, first, the basic cuckoo search algorithm was applied to optimize the ANN model and then two modifications (*P*_*a*_Ctrl-CS and Multipop-*P*_*a*_Ctrl-CS) of the cuckoo search were proposed. These modifications were designed to improve the exploration and exploitation of the algorithm and its ability to achieve better convergence. Control of the *P*_*a*_ parameter in *P*_*a*_Ctrl-CS was achieved by setting this parameter to a maximum value in the initial stage to gain more exploration and by decreasing this parameter during the search process to gain more exploitation until the algorithm reached final convergence with the minimum value of *P*_*a*_. Furthermore, a master-leader-slave multi-population strategy was embedded in Multipop-*P*_*a*_Ctrl-CS to improve the convergence ability of the algorithm. In this strategy, the slaves with aid of leader guidance provided good exploration and the master had the role of providing the algorithm with more exploitation. Based on extensive evaluations it is concluded that the Multipop-*P*_*a*_Ctrl-CS algorithm has the ability to outperform other recent algorithms in the literature in five out of six classification problems. The algorithm also demonstrated better performance on two tested time series prediction problems. We believe that the superiority of the results is due to the fine balancing between exploration and exploitation in Multipop-*P*_*a*_Ctrl-CS provided by *P*_*a*_ control and the master-leader-slave multi-population. Finally, the proposed methods were applied to real-world data for water quality prediction. The promising results for both benchmark and real-world data motivate us to improve this method in future work.
